# Surface Potential/Charge Sensing Techniques and Applications

**DOI:** 10.3390/s20061690

**Published:** 2020-03-18

**Authors:** Songyue Chen, Hepeng Dong, Jing Yang

**Affiliations:** Department of Mechanical and Electrical Engineering, Xiamen University, Xiamen 361005, China; 19920171150927@stu.xmu.edu.cn (H.D.); jingyang@stu.xmu.edu.cn (J.Y.)

**Keywords:** surface potential, surface charge, nanowire, nanopore, zeta potential

## Abstract

Surface potential and surface charge sensing techniques have attracted a wide range of research interest in recent decades. With the development and optimization of detection technologies, especially nanosensors, new mechanisms and techniques are emerging. This review discusses various surface potential sensing techniques, including Kelvin probe force microscopy and chemical field-effect transistor sensors for surface potential sensing, nanopore sensors for surface charge sensing, zeta potentiometer and optical detection technologies for zeta potential detection, for applications in material property, metal ion and molecule studies. The mechanisms and optimization methods for each method are discussed and summarized, with the aim of providing a comprehensive overview of different techniques and experimental guidance for applications in surface potential-based detection.

## 1. Introduction

Surface potential sensing techniques measure the surface charge density over the solid surface or solid–liquid interface. Most recent developments in sensing techniques focus on the latter situation due to the difficulties associated in charge detection in liquid. The surface potential over the solid–liquid interface is vital for a variety of physicochemical related applications, such as chemical, engineering, biological, and even production processes, including sensing [1,2,3], catalysis [4], corrosion prevention [5], cell adhesion and aging study [6,7], nanoparticle separating [8], pulp toughness [9] and so on. In the presence of an electrical double layer (EDL) when immersing solid into liquids, the target chemical or biological inputs are transformed into electrical signals, referred as surface potential or surface charge. Surface potential *ϕ*_o_ has a linear relationship with surface charge density as *σ* = *C*_dl_*ϕ*_o_, where *C*_dl_ is the double layer capacitance that is determined by the ionic strength and ionic valence. Surface potential can be modulated by adsorption of target molecules, e.g., chemical concentrations (pH [10,11,12], metal ion concentration [13,14], etc.), biological species (DNA [15,16,17], protein [18], etc.), electrical modulation (reference electrode) and physical movement (flow velocity [19,20]). In other words, those variables that induce potential or charge change can be detected through surface potential or charge measurement.

**Figure 1:**
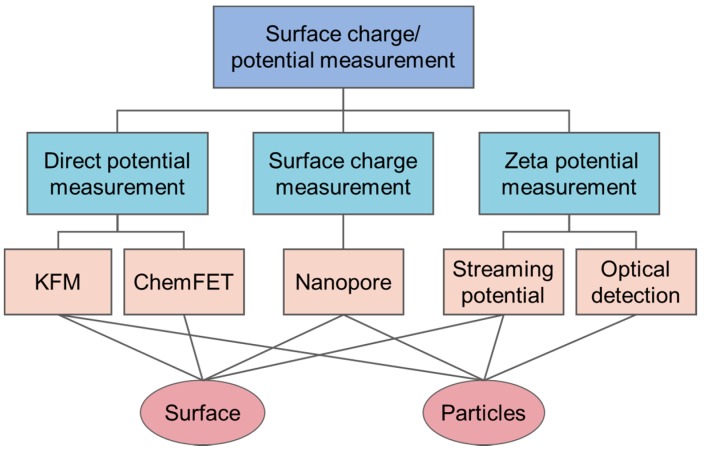
Surface charge/potential measurement techniques.

In this review, surface potential/charge detecting methods are categorized into three groups for characterizing macroscopic surface and/or micro/nano particles (as shown in Figure 1): direct potential measurement with Kelvin probe force microscopy and chemical field-effect transistors (chemFETs), surface charge measurements with nanopore sensors, and zeta potential measurements with streaming potential measurements and optical detection techniques.

## 2. Direct Surface Potential Measurement

### 2.1. Kelvin Probe Force Microscopy (KFM)

Kelvin probe force microscopy (KFM) is a tool that can perform nanoscale imaging of surface potential for a variety of materials [21] at a sub-nanometer resolution [22]. It is an atomic force microscope (AFM)-based instrument that measures the contact potential difference (CPD) between the sample surface and the tip, with *V*_CPD_ = (*φ*_tip_ − *φ*_sample_)/*e*, where *φ*_tip_ and *φ*_sample_ are the work functions of the tip and the sample, respectively, and *e* is the elementary charge. When the AFM tip approaches the sample surface, electrical potential is generated due to the difference in Fermi levels. In 1991, Nonnenmacher initiated the technology for measuring the contact potential between different metal materials [21]. Thereafter, KFM has been widely used to characterize the localized surface potential of metals, semiconductors [23,24,25], TiO_2_ [26], organic materials [27,28], biomaterials [29] in polar liquids and, recently, in non-polar solutions as well [30]. There are two main operation modes: oscillation amplitude modulation (AM) mode and oscillation frequency modulation (FM) mode [22]. AM mode measures the direct force between the tip and sample [31]; FM mode detects the force gradient [32], which gives a higher spatial resolution. The AM mode has a spatial resolution of, typically, 25 nm and a potential resolution of 5 mV [22]. The FM mode can reach sub-nanometer resolution and has a potential resolution of 10–20 mV [22]. 

**Figure 2:**
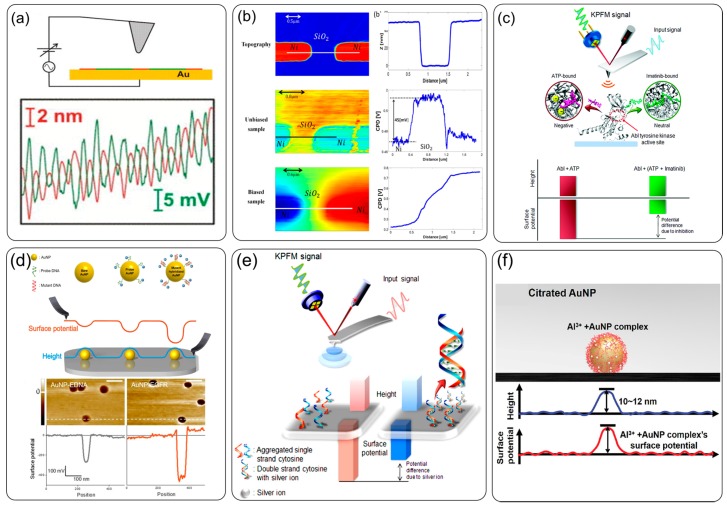
Kelvin probe force microscopy (KFM) for surface potential images: (**a**) schematic diagram of the detection in air (top graph), and in the bottom graph, the red line represents the topography for fibril, and the green line represents the surface potential [30]; (**b**) the morphology and contact potential difference for metal contacts on SiO_2_ surface for unbiased and biased sample [34]; (**c**) recognition of single-molecule interaction between protein and small ligands [35]; (**d**) DNA on a gold nanoparticle (AuNP) for mutation study by comparing the height and surface potential signals [36]; (f) single-molecule detection of avidin molecules and DNA [37]; (**e**) Ag^+^ ion adsorption to aptamers [39]; and (**f**) Al^3+^ ion detection on citrated AuNPs [40].

KFM is commonly used for solid surface inspection. When combined with an optical fiber probe, the photoexcitation effect on surface potential of Alq_3_ thin films can be measured [33]. Stone et al. [30] demonstrated qualitative estimation of fibril surface charge with different functional chemical groups after exposure to water through potential measurement by KFM (Figure 2a). Cohen et al. [34] obtained potential measurements for metal contacts on SiO_2_ and showed the effect of voltage bias, as shown in Figure 2b. 

Besides solid and wet surfaces, KFM are also applied in ion and biomolecule detection with localized surface chemistry design. Park et al. [35] demonstrated the precise recognition of biomolecule interaction with KFM, which can quantitatively describe single-molecule interactions, such as protein kinase–ATP interactions, as shown in Figure 2c. Jang et al. [36] reported a highly sensitive mutation detection with a gold nanoparticle (AuNP)-based platform (Figure 2d) by evaluating the surface potential change of each AuNP on silicon substrate, during probe DNA modification and target DNA hybridization, with a detection limit of 3.3 pM. Leung et al. [37] showed the single-molecule detection of avidin molecules and DNA, with a resolution of five basic amino acids. They based the expected charge pattern measurements on individual DNA molecules and proteins after further refinements. The metal ion detection is based on the surface potential change at ionic adsorption, e.g., Hg^2+^ ion detection by formation of thymine–Hg^2+^–thymine at a detection limit of 2 fmol [38], Ag^+^ ion detection by formation of cytosine–Ag^+^–cytosine at a detection limit of 20 fmol, as schematically shown in Figure 2e [39], and Al^3+^ ion detection by exposure to citrated AuNPs at a detection limit of 2 amol, as shown in Figure 2f [40]. The material difference between the substrate and nanoparticles favors specific surface chemistry for potential measurements—e.g., thiol chemistry works on Au, but not on silicon substrate.

Compared to other measurement techniques, KFM has excellent spatial resolution and relatively high potential sensitivity. However, measurement of the absolute surface potential using KFM needs a reference work function, which requires the calibration of the KFM probes [41]. Performing the necessary exchange of the two samples under the KFM probe will reduce the accuracy of the measurement [41]. When measuring a surface, KFM can only measure changes in the average surface potential of the molecule. Sudden changes in terrain height reduce the accuracy [22,42]. Besides, the time cost for such a measurement is high and the measurements can only work in non-conducting environments. More recently, another AFM-based potential detection technique was developed for electrostatic force imaging by analyzing the resonant frequencies. Specifically, electrostatic force microscopy operated in multifrequency mode, provides a promising technique for improving the spatial resolution and time resolution [43], and can be applied to obtain electrostatic and topographic images simultaneously [44,45].

### 2.2. Chemical Field Effect Transistor (ChemFET)-Based Potential Sensing

**Figure 3:**
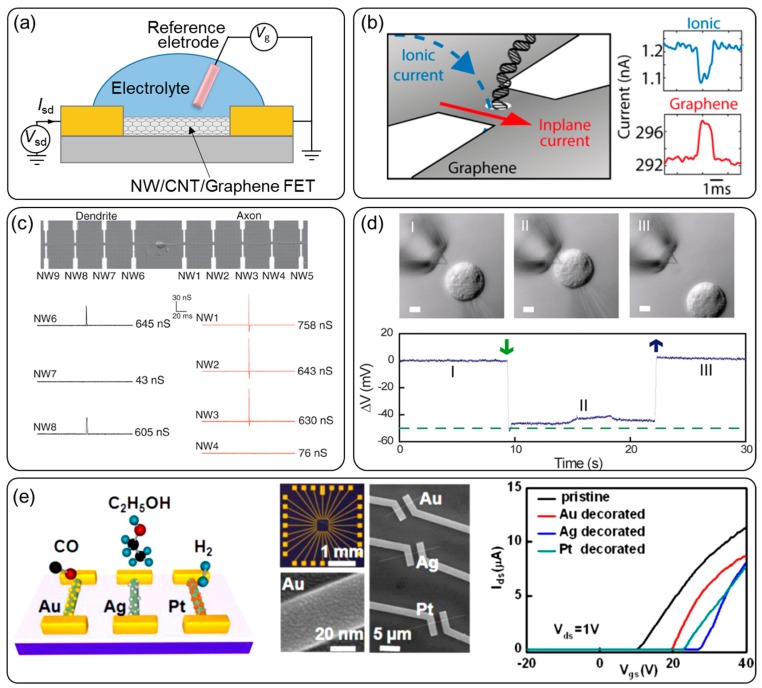
NanoFET configuration and applications: (**a**) schematic configuration of NanoFETs, (**b**) DNA translocation event detection with a graphene nanoribbon and a nanopore simultaneously [62], (**c**) extracellular neuronal signal detection with high-density nanowire arrays [64], (**d**) intracellular potentials recording with a Si NWs [65], (**e**) Si NW arrays for gas composition sensing [67].

When a chemFET is immersed in an electrolyte, the electrostatic gating through the liquid and gate dielectric layer, generally named liquid-gating, modifies the carrier mobility in the transistor, as illustrated in Figure 3a. When the surface charge density on the gate changes, the surface potential can be measured by the device conductivity change. Materials for such FET devices include semiconductors, e.g., ion sensitive field effect transistor (ISFET) and Si nanowires (NWs) [12,46], nano ribbon [18,47], carbon nanotubes (CNTs) [15,48], and graphene [1,10,49]. 

The surface potential change induced conductivity change is generally monitored through a constant current mode [50], which is commonly used for ISFET, a constant voltage mode [12,51], a threshold voltage shift scan [15,52,53], the ion-step method [14,54], or a Dirac voltage shift scan (for graphene [55,56]). A constant current mode is generally used in ISFET where the current value is relatively high, and a feedback voltage is applied to the gate voltage to maintain a constant current in the transistor. The change in feedback voltage is therefore regarded as the surface potential change. A constant voltage mode is mainly used in nanostructured field effect transistors (nanoFETs), e.g., NW, CNT, Graphene, where the current is generally small and the surface potential induced conductivity change is monitored by electric current. Threshold voltage scan can be applied in most device conditions by applying a scanning of gate voltage and finding a threshold voltage recording to the characteristics of the gate voltage-current curve. For graphene, the corresponding variable is Dirac voltage, where there are equal amounts of electrons and holes. Ion-step method is a stimulus-response method that is less sensitive to drift [14]. An ion-step from low to high ionic strength solution is applied to the gate oxide. A sudden increase of double layer capacitance *C*_dl_ is expected and a change in current can be measured due to the reduction in surface potential, since *ϕ*_o_ = *σ*_o_/*C*_dl_, where *σ*_o_ is the surface charge density.

ChemFETs, especially nanoFETs, for surface potential detection are applied extensively in different fields. ISFET sensors were initially designed based on the proton sensitivity of the gate oxide–electrolyte interface, and later for membrane-covered ISFETs for various types of target detection [57,58], e.g., charged protein sensing with an immobilized antibody [59] ordetection of proton release from protein phosphorylation by catalysis [60]. The development of nanoFET brought FET sensors higher spatial resolution and sensitivity. Si NW biosensors are used for DNA detection and they can achieve a 1fM level by enhancing the signal with rolling circle amplification [61]. The Dekker’s group demonstrated a probed DNA translocation event with a graphene nanoribbon where a nanopore was also fabricated [62]. Both their nanoribbon and nanopore current signals are recorded for comparison (Figure 3b). They can be used to construct a localized probe for extracellular and intracellular biochemical potential measurements [63,64,65]. The Lieber group detected extracellular neuronal signals with high-density NW arrays [64], as shown in Figure 3c, which can measure the rate, amplitude, and shape of potential signal propagation along single axons and dendrites. They later fabricated a kinked Si NW for intracellular potential recordings after modification with phospholipid bilayers (Figure 3d) [65]. Besides, the fabrication process for ChemFETs is amenable to minimization and integration. NanoFETs allow array fabrication for the multiplexed sensing of biomoleculars [66] and gas compositions [67], which are performed at the chip level. Zheng et al. designed a real-time multiplexed electrical detection of four different cancer protein markers in serum samples [66]. Zou et al. decorated the NW surface with three different metal nanoparticles [67], Au, Ag and Pt, which allow the simultaneous detection of CO, C_2_H_5_OH, and H_2_.

The surface potential sensitivity of chemFET can be modulated by a few parameters, including the fabrication parameters (e.g., doping, size) [68,69], device operation [52,70], the interface material [51,71], electrolyte ionic strength [72], surface chemistry [73], etc. Doping and size have a significant impact on the charge sensitivity. The reduction in size gives nanoFETs very large surface-to-volume ratio and makes them extremely sensitive to the surface properties. Moreover, Park et al. [74] demonstrated that, by decreasing the impurity doping concentration, the sensitivity increased greatly compared to reducing the device diameter. At the operation level, the tuning of the device in the depletion region and at a higher frequency (e.g., 30 Hz input voltage compared with DC voltage) can optimize the signal-to-noise ratio and the limit of detection [75]. By coupling the back gate with the liquid gate [76], the signal-to-noise ratio in both subthreshold regimes and above-threshold regimes can be improved.

Interface materials with improved dielectric property increase sensitivity. Chen et al. [51] deposited Al_2_O_3_ gate oxide on Si NWs with atomic layer deposition, and showed near-Nernstian pH sensitivity and superior repeatability. Bashir et al. [71] added a layer of high-k hafnium oxide, to reduce the dielectric thickness and the leakage current, and demonstrated an improved biomolecule sensitivity. Additionally, properly designed surface chemistry, e.g., with additional polyethylene glycol modification, extends the Debye length, and therefore increases the sensitivity of NW sensors in high ionic strength solutions [77]. Masood et al. [78] selectively functionalized alkenyl monolayers on the silicon surface with a carbon–silicon alkyl, which not only reduced the gate thickness, but also eliminated the fixed oxide charge, and showed dramatic improvements in the device performance and detection sensitivity. By controlling distance of the surface charge layer to the sensor surface through molecular engineering [79] or by bringing the probe in closer proximity to the sensor surface through probe size reduction [80], the device sensitivity can be modulated. The field effect gets stronger when the charged target molecular layer moves closer. Park et al. [59] reported an interfacial charge regulation method by protein-blocking layers on ISFET for direct measurements in serum. Bhattacharyya et al. [81] proposed a local electrostatic method to tune the Debye length, forcing the double layer ion concentration to match the bulk.

## 3. Surface Charge Measurement

The surface charge measurement in liquid can be realized with solid-state nanopore sensors, including polymer nanopores and inorganic nanopores or nanochannels [2]. Nanopores take advantage of the electrostatic effects inside nano-confined space in the presence of surface charges, which gives them high sensitivity and new sensing mechanisms [82]. The change in surface charge regulates the ionic conductance of nanopores through pH [83], divalent cations and anions [84], ionic concentrations [85], temperature [86], surface chemistry and biomolecules [87], gas [88], salt gradient and voltage polarity [13], and can be characterized by the current–voltage (*I–V*) curve due to the conductance change that is related to the inner wall surface charge regulation, or the instant current change related to the passing of charged particles through nanopores. The non-uniform distribution of surface charge inside the nanopore greatly affects the current rectification ratio. One of the required condition for surface charge detection is low background ionic concentrations [82,89] in order to minimize the conductance contribution from bulk ions. Since the nanopore diameter has a large influence on the sensitivity [82], smaller nanopores favor charge detection that benefits from electric double layer overlap.

**Figure 4:**
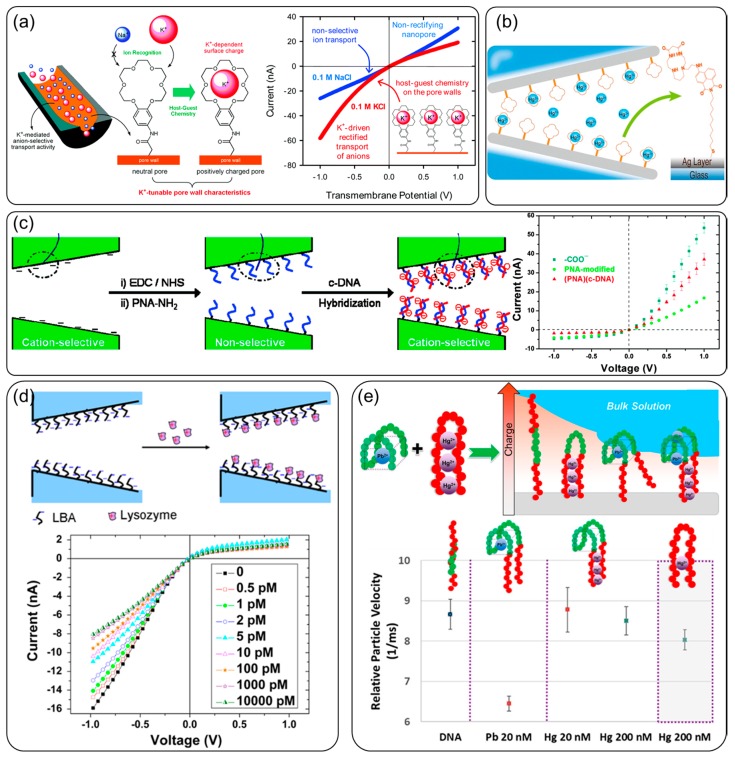
Nanopores for surface charge detection: (**a**) crown ethers chemistry for specific adsorption of K^+^ ions and the current rectification change [90]; (**b**) macrocyclic dioxotetraamine derivative functionalization for Hg^2+^ ion determination [91]; (**c**) DNA detection based on PNA–DNA recognition, and the current–voltage (*I–V*) curve change [93]; (**d**) lysozyme detection with aptasensor [94]; (**e**) charged particle detection based on aptamer velocity difference for different heavy metal ions [95].

For inner wall surface-based charge regulation, the stimulus from the solution that caused the charge change alters the *I–V* curve characteristics. By designing proper surface chemistry, metal ion concentrations can be measured through the current rectification ratio, e.g., crown ethers chemistry for the specific adsorption of K^+^ ions (Figure 4a) [90], macrocyclic dioxotetraamine derivative functionalization for Hg^2+^ ion determination at a level of 10 pM (Figure 4b) [91], and polyglutamic acid for repeatable Cu^2+^ ion sensing [92]. The adsorption of positively charged metal ions increases the positive charge and, therefore, changes the current rectification ratio. Taking advantage of the biomolecule recognition reaction, e.g., DNA–peptide nucleic acid (PNA), nanopores are used for the sequence specific detection of single-stranded DNA, as shown in Figure 4c [93]. The signal was enhanced by diminishing the channel surface charge with uncharged PNA probes. The hybridization of DNA molecules to the probes increased the negative surface charge. A glass nanopore-based aptasensor was used for lysozyme detection with a sub-pM detection limit [94]. In addition, nanopore sensors can monitor the mobility of charged particles. Figure 4e shows the recognition of different heavy metal ions by monitoring particle velocity while passing an aptamer through the nanopores [95].

Although there is a broad amount of research attention paid to solid-state nanopore sensors, there are still remaining questions for the surface charge regulation mechanism inside nanopores and limited applications for stable and repeatable sensing. It is known that there is a significant deviation in pH inside the nanopore from the bulk [96]; however, the regulation mechanism is not clear while performing surface chemistries. Furthermore, the addition of buffer solution also has a significant influence on the charge sensitivity at a low salt concentration and pH [97]. More importantly, the relationship between the surface charge and the *I–V* characteristics of the nanopore is not yet clear and is difficult to obtain. Therefore, this calls for a systematic understanding of surface charge sensing inside confined spaces.

## 4. Zeta Potential Measurement

Zeta potential is the electric potential of the slipping plane in the electric double layer with the moving of liquid or solid particles, and is related to the surface charge [98,99]. It is an important parameter describing the behavior of the solid–liquid interface charge, it is affected by solid material properties and liquid phases [100,101], and it represents the key physicochemical surface properties in various fields from electrochemistry to pharmaceuticals [99,102,103]. The zeta potential measurement of a solid wall in solution depends on the streaming potential or electroosmotic mobility measurement technique [104]. For colloids or nanoparticles in suspension, zeta potential is a very important parameter, which is closely related to suspension stability and particle surface morphology. Therefore, zeta potential measurement is widely used in product stability studies and surface adsorption studies [105].

### 4.1. Streaming Potential Measurement

**Figure 5:**
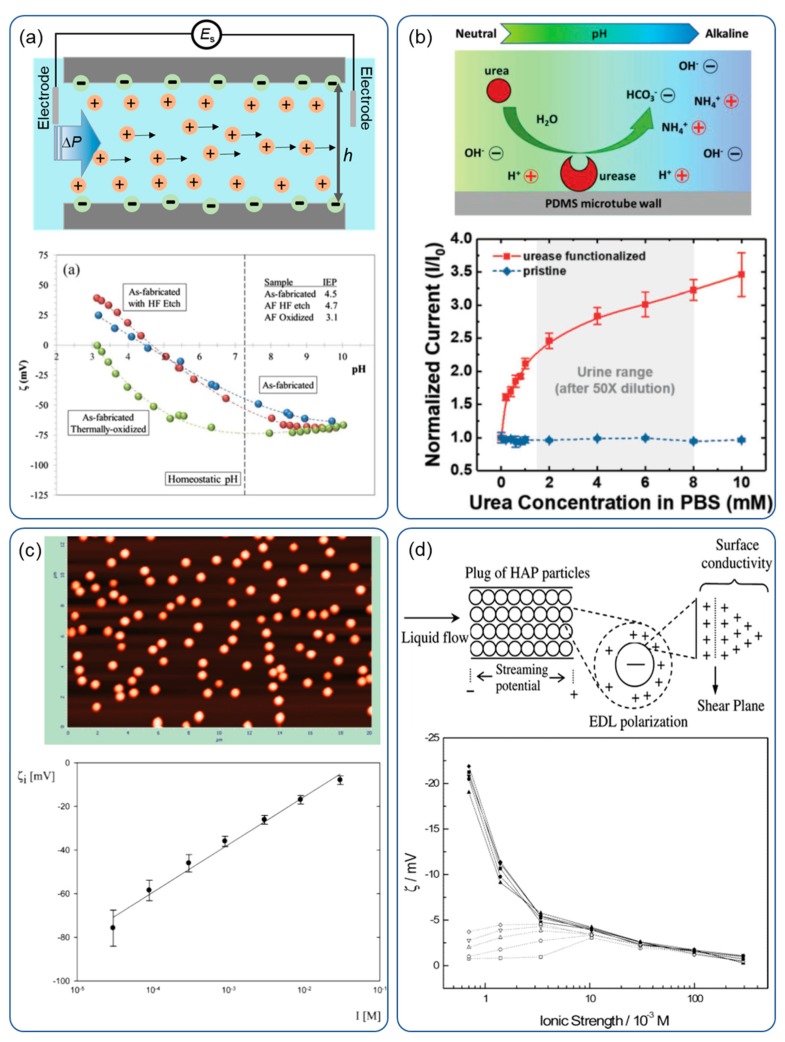
Streaming potential/current measurement for different applications. (**a**) A schematic setup (top) and characterization of surface treatment to silicon nitride surface (bottom) [109]; (**b**) schematic of urea hydrolysis catalyzed by the urease immobilized on microtube wall (top), and current output increases with increasing urea concentration from 0.0–10.0 mM (red line). The current output is independent of urea concentration without urea (blue line) [114]; (**c**) AFM (scan width 20 μm) of latex particle monolayers on mica (top), and the zeta potential at different MgCl_2_ concentration (bottom) [113]; (**d**) schematic diagram of a plug for detecting hydroxyapatite particles with flow potential (top), and relationship between the zeta potential and KCl ion concentrations (bottom) [115].

Flow potential technology has been widely used in planar polymer and glass surfaces to study the electrical properties of solid–liquid interfaces in parallel plate microchannels [106]. For steady incompressible and laminar flow, the streaming potential *E*_s_ can be related to the ζ -potential via [107]:

(1) $$\frac{E_{S}}{∆P}=\frac{\varepsilon_{r}\varepsilon_{0}\zeta }{\mu }\frac{1}{\left(\lambda_{b}+2\lambda_{s}/h\right)}$$

where Δ*P* is the pressure difference, *h* is the channel height, *λ_b_* is the bulk conductivity, and *λ_s_* is the surface conductivity [107]. In order to get a measurable streaming potential, a low bulk conductivity is required, which means a low ionic strength liquid. The detection mechanism is schematically shown in the top image of Figure 5a. 

Streaming potential or current measurement has been widely used to characterize charged bulk materials or particles, e.g., biospecific interaction study [108], inorganic material surfaces [109], membranes [110], wood [111], natural and man-made fibers [112], and colloid particles [113]. The bottom image of Figure 5a shows surface treatments, e.g., etching and oxidation, had a profound influence on the zeta potential of the silicon nitride surface [109], where the oxidized nitride surface might resist bacterial colonization due to its extreme negative surface charge. Li et al. [3] designed a DNA detection system based on DNA–PNA hybridization inside a microchannel, measured with a streaming potential analyzer, and obtained a detection limit of 10 nM. Yu et al. [114] developed a self-powered urea sensor by immobilizing catalytic enzymes on the microfluidic channel. The fluid pH increased when hydrolyzing urea into ions, which changed the measured streaming current. 

In order to characterize the zeta potential of particles, the immobilization of particles into a substrate is required. Adamczyk et al. [113] covered mica with monodisperse latex particles, and measured the streaming potential with the addition of different salt solutions. Figure 5c shows the AFM image of latex particle monolayers on mica, and the reduction in the zeta potential of the surface at increased MgCl_2_ ionic strength. Another way to measure the zeta potential of particles is to pack particles inside a plug, then measure the streaming potential by the flow of liquids through the plug [115]. Figure 5d shows the design for such a device, for detecting hydroxyapatite particles and the zeta potential relation, with a KCl concentration [115]. 

### 4.2. Optical Detection

Besides the streaming potential method, the zeta potential of particles can also be measured indirectly through electrophoretic motion [116], where electrophoretic light scattering (ELS), acoustics and electroacoustics are commonly used methods. Among the three methods, ELS is commonly chosen for many applications due to its sensitivity, accuracy and versatility [117,118] in determining the zeta potential of suspended particles. However, the classic ELS uses transmitted light and receives scattering at a small angle (typically 8°–30°), so it cannot be used for turbid samples because incident light cannot penetrate the sample [103].

**Figure 6:**
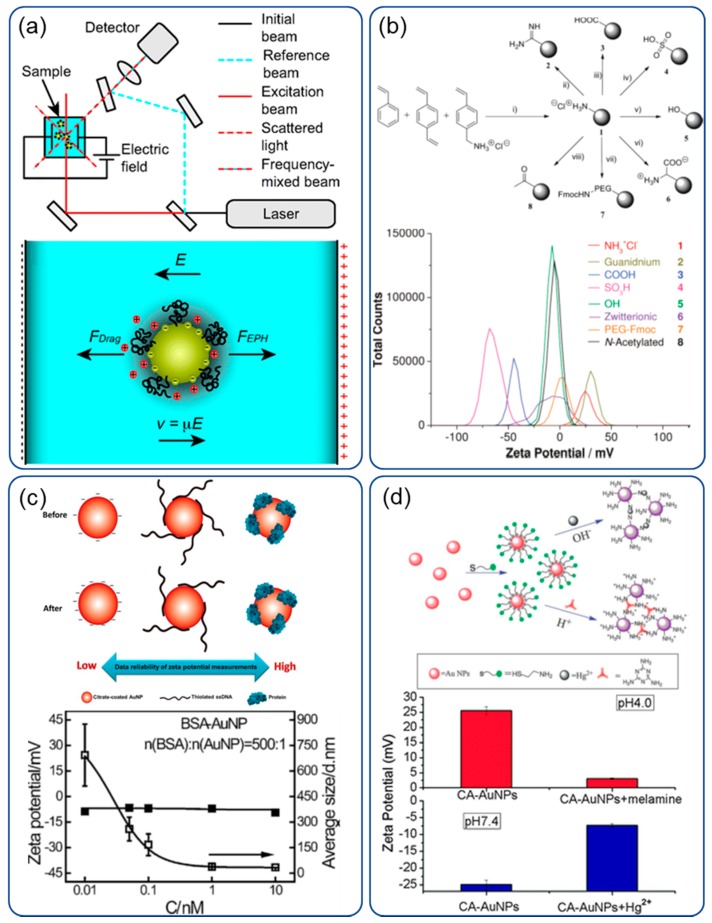
Electrophoretic light scattering for zeta potential measurements: (**a**) the light path for the detection system and the force analysis of particles under detection [119]; (**b**) zeta potential difference for various surface chemistries, e.g., amino, carboxyl and poly(ethylene glycol) and so on [123]; (**c**) bovine serum protein detection with AuNPs [124]; (**d**) surface chemistry for Hg^2+^ ion detection and a resultant reduction in zeta potential for Hg^2+^ ion adsorption [125].

Since molecules and particles are electrophoretic under the action of an applied electric field, their velocity is directly related to the amount of charge, and can be measured using laser coherence technology—specifically, the phase analysis light scattering method (M3-PALS). Figure 6a,b show a typical setup [119], where the laser beam is divided into a reference beam and an excitation beam. The excitation beam passes through the sample and the electric field is applied to induce particle electrophoresis. Moving particles scatter incident laser light, which causes a frequency shift from the excitation laser. The frequency shift is proportional to the speed of the particles, which is called Doppler shift [120]. The frequency difference is then measured by the ELS detector. According to Henry’s equation and the light scattering Doppler shift theory, the zeta potential ζ of the particles can be calculated with [99]:

(2) $$\varsigma =\frac{3\eta \lambda \Delta f}{4\varepsilon EF\left(ka\right)\sin\left(\frac{\theta }{2}\right)}$$

where *λ* is the wavelength of incident light, Δ*f* is the Doppler frequency shift; *ε* is the dielectric constant of the dispersant; *E* is the electric field strength; *n* is the refractive index of the solution; F(*κ*a) is the Henry function and 1/*κ* is the Debye length, *a* is the radius of spherical particles; *η* is the viscosity; *θ* is the scattering angle.

The advantages of measuring zeta potential with ELS include minimal sample preparation, the analysis of large numbers of particles to provide good statistical results, and the use of disposable capillaries to prevent cross-contamination between samples. However, ELS is only suitable for uniform samples made with materials with the same optical characteristics [121]. Sample concentration also has a significant impact on the zeta potential measured with ELS [122]. Low concentrations can greatly reduce the signal-to-noise ratio and cause noisy and inconsistent results. In the contrary, high concentrations cause multiple scattering effects and particle interactions [98]. The optical configuration used in a typical laser Doppler instrument (such as Zetasizer Nano) calls for an optically transparent sample [99]. Therefore, in order to accurately measure the zeta potential, it is often necessary to dilute samples to reduce scattering. Another solution is to use unique electrodes that are conductive but transparent to incident and scattered light, which renders a shorter optical path length at a smaller scattering angle (about 35°), so as to avoid the interference of Brownian expansion [103]. One other way to measure the electrophoretic mobility of samples with a higher concentration is to reduce the path length of the cells, which increases the transmittance of the laser through the sample. Although such a cell can perform electrophoretic mobility measurements on a concentrated sample, the conversion to a zeta potential and subsequent interpretation of the obtained data is not easy. Kaszuba et al. [99] discussed electrophoretic mobility measurements on a high-concentration turbid sample with a new shortened cell, and found that, for the two sample types studied (titanium dioxide and polyurethane dispersion), the electrophoretic mobility showed a gradual decrease with increasing sample concentration.

Both nanoparticles and microparticles can be used for the zeta potential analysis of their surface modification [123]. Figure 6b shows the zeta potential difference for the amino, carboxyl and poly(ethylene glycol) functionalized surface. Furthermore, this can also be used to monitor the formation of nanoparticles with protein [126]. Based on such a mechanism, Wang et al. [124] presented biomolecular conjugated gold nanoparticles for ultra-sensitive protein quantification, as shown in Figure 6c. Bovine serum albumin on gold nanoparticles obtained a single-molecule resolution. Ma et al. [125] proposed a zeta potential sensing mechanism based on the electrostatic attraction of melamine and the formation of an N–Hg^2+^–N structure for the gold nanoparticles in the presence of Hg^2+^ ions. Figure 6d outlines the sensing mechanism and the measured results. The decrease in negative charge on cysteamine-modified gold nanoparticles indicates the tendency of Hg^2+^-caused aggregation.

## 5. Conclusions and Outlook

In this review, we summarized the surface potential/charge measurement techniques for monitoring the electric status of macroscopic planes and micro/nanoparticles, for applications in surface characterization, metal ion and biomolecule sensing. The detection mechanisms are discussed with optimization strategies. Both surface chemistries and sensing mechanisms have significant impacts on the sensitivity. The presence of nanosensors, e.g., nanowires and nanopores, bring versatility, but also new challenges. Table 1 shows a comparison between the different detection techniques.

Further optimization of sensing techniques should focus on improving the sensitivity through the surface chemistry, sensing technique and analyzing method. Surface functionalization not only offers opportunities for a variety of targets, but also for complicated detection environments, e.g., direct measurements in serum with ISFET and protein blocking layers [59]. Finally, the combination of multiple techniques could benefit each other and offer robust and reliable methods to validate reactions [62,126]. We hope this review will provide cross-learning and new ideas for the development and applications of surface potential/charge detection techniques.

## Figures and Tables

**Table 1:** Comparison of surface potential/charge detection techniques.

Detection Technique	KFM	ChemFET	Nanopore	Streaming Potential	Optical
Measurand	Surface potential	Surface potential	Surface charge	ζ-potential	ζ-potential
Detection signal	Potential difference	Current	Ionic current	Streaming potential/current	Frequency shift
Detection conditions	Non-conducting liquid and air	Both liquid and air	Low bulk conductivity liquid	Low bulk conductivity liquid	Liquid
Sensitivity	5 mV	1.5 mV	-	Sub-mV	Sub-mV
Portability	Non portable	Minimizable	Minimizable	Non portable	Portable
Application	Localized surface potential characterization; Ion detection; Biomolecule detection	Ion sensing; Biomolecule sensing; Cellular potential detection; Gas sensing	Ion sensing; Biomolecule detection; Gas sensing	Surface characterization; Ion sensing; Biomolecule detection	Ion sensing; Biomolecule detection;
Pros and cons	Sub-nm spatial resolution; Time consuming	nm scale resolution; Fast	Difficult signal conversion to surface charge; Fast	Wide applications; Medium speed	Minimal sample preparation; Require uniform sample; Fast
References	[22,33,34,35,36,37,38,39,40]	[51,52,53,54,55,56,57,58,59,60,61,62,63,64,65,66,67,75]	[83,84,85,86,87,88,89,90,91,92,93,94,95]	[107,108,109,110,111,112,113,114,115]	[116,117,118,119,120,121,122,123,124]
